# Supplementary material for: Conserved glucokinase regulation in zebrafish confirms therapeutic utility for pharmacologic modulation in diabetes
Source: Commun Biol. 2024 Nov 23;7:1557. doi: 10.1038/s42003-024-07264-5 (PMC11585571; doi:10.1038/s42003-024-07264-5)
Supplement: Supplementary file 1 — Supplementary Information [file 42003_2024_7264_MOESM1_ESM.pdf]

Conserved glucokinase regulation in zebrafish confirms therapeutic utility for pharmacologic modulation in diabetes

Nicole Schmitner[1], Sophie Thumer[1], Dominik Regele[1], Elena Mayer[1], Ines Bergerweiss[1], Christian Helker[2,3], Didier Y.R. Stainier[2], Dirk Meyer[1], Robin A. Kimmel[1]

[1]Institute of Molecular Biology, Center for Molecular Biosciences Innsbruck (CMBI), University of Innsbruck, Innsbruck, Austria, [2]Max Planck Institute for Heart and Lung Research, Bad Nauheim, Germany, [3]present address: Philipps-University Marburg, Marburg, Germany

Supplemental Material

Supplementary Table 1

gck_HA1_GFP_fw	CACATTCAAACTTTAGCAAATCATCCTACAATAGTTTCAAGA GACCTGCAACCATGGTGAGCAAGGGCGAGGAG
gck_HA2_GFP_kanR_rev	GACTCAAAGTCGCTCGGTATCCTCTGTCTACGGGCTGAAGTA AGACACGGTTCCAGAAGTAGTGAGGAG
gck_HA1_LUC2_fw	CACATTCAAACTTTAGCAAATCATCCTACAATAGTTTCAAGA GACCTGCAACCATGGAAGATGCCAAAAACAT
gck_HA2_LUC2_kanR_rev	GACTCAAAGTCGCTCGGTATCCTCTGTCTACGGGCTGAAGTA AGACACGGTGACCATGATTACGCCAAGC
Gck probe f	CTCCTCCAGCGCAGCATAAA
Gck probe r	CAAAGTAAATTCAGACCCTGTGGA
Ef1α qPCR f	TCTCTACCTACCCTCCTCTTGGTC
Ef1α qPCR r	TTGGTCTTGGCAGCCTTCTGTG
Ins qPCR f	GCCCAACAGGCTTCTTCTACAAC
Ins qPCR r	GCAGATTTAGGAGGAAGGAAACCC
Gck qPCR f	GACCGGCACAGGTGAAATGTTG
Gck qPCR r	TCCAAATCCTCGTGACGCACTG
Hk qPCR f	ACTTTGGGTGCAATCCTGAC
Hk qPCR r	AGACGACGCACTGTTTTGTG
Pklr qPCR f	GGTTCTTCCGCTCAGGTGAT
Pklr qPCR r	CCCAGGAGGCTGTCAATCAA
Glut2 qPCR f	TTAACAGGCACGCTCGCTCT
Glut2 qPCR r	TTCATGCTCTGTGCCATTTCC

Supplemental Methods

Bioinformatic analysis

To compare overall expression and isoform usage of gck between liver and pancreas cells, published RNASeq raw read data in fastq format were retrieved from the European Nucleotide Archive. Data from ref 1, stored under the accession PRJEB10140, were used for pancreatic endocrine cells. Samples archived under the accessions SAMEA3498616, SAMEA3498617, SAMEA3498618, SAMEA3498619 and SAMEA3498620 were used to analyze the transcriptome of β-cells. Samples archived under the accessions SAMEA3498621, SAMEA3498622, SAMEA3498623, SAMEA3498624, and SAMEA3498625 were used to analyze the transcriptome of α-cells. Samples archived under the accessions SAMEA3498626, SAMEA3498627, SAMEA3498628, SAMEA3498629 were used to analyze the transcriptome of δ-cells. The following transcriptome data of zebrafish livers (4-6 dpf) from ref 2 were used to characterize gck expression in the liver: SRR19671831 and SRR19671832 (4 dpf); SRR19671827 and SRR19671828 (5 dpf); SRR19671826 and SRR19671829 (6 dpf).

Quality control, filtering, and read trimming were performed using fastp 0.23.2 [3] in paired end mode, using the parameters detect_adapter_for_pe, cut_front, cut_tail, trim_poly_g, length_required 50, and overrepresentation_analysis. BAM files for transcript quantification were obtained by mapping the filtered reads using an index built with the zebrafish genome (GRCz11, primary assembly) and the Lawson lab transcriptome annotation V4.3.2 [4] using STAR (2.7.10a_alpha_220314, [5]). Mapping with STAR was performed using the parameters quantMode TranscriptomeSAM, outFilterType BySJout,

outFilterMultimapNmax 20, alignSJoverhangMin 8, alignSJDBoverhangMin 1, outFilterMismatchNmax 999, outFilterMismatchNoverReadLmax 0.04, alignIntronMin 20, alignIntronMax 1000000, alignMatesGapMax 1000000. RSEM (v1.3.1, [6]), together with an RSEM reference index built against the same genome assembly and transcriptome annotation as above, was used to obtain transcript counts and isoform usage percentages for all genes. Some libraries for pancreatic endocrine cell samples were sequenced multiple times. Transcriptome BAM files from these libraries were merged using samtools merge [7]. The transcriptome aligned reads in BAM format were used as input for rsem-calculate-expression in paired end mode with default parameters.
Isoform usage percentages for gck transcripts were obtained directly from the RSEM output files. Log-normalized counts per million (CPM) on gene or transcript level were obtained using edgeR [8] in R (v4.4.0) [9], according to the code provided in the edgeR vignette. Briefly, raw count matrices from RSEM were imported using tximport [10]. For gene level analysis, transcript counts were aggregated to gene counts. Counts for each transcript were scaled by length. Effective library sizes were calculated from scaled counts and combined with the length factors. Offsets for a log-link Generalized Linear Model (GLM) were derived and used to create a DGEList object from the scaled counts using the DGEList function. Counts per million (CPM) were computed with these offsets and log-transformed for visualization.

Amino acid alignment

Amino acid sequences from mouse and human GCK isoforms (HK4 ISO1 and ISO2) were retrieved from https://www.ncbi.nlm.nih.gov/protein/. Amino acid sequences from zebrafish were translated from nucleic acid sequences in Benchling (https://benchling.com.). Sequences were aligned using ProteinBlast (https://blast.ncbi.nlm.nih.gov/Blast.cgi). Alignments were then loaded into Jalview and modified.

Antibody staining of adult islets

Animals were euthanised on ice and pancreatic islets were dissected, fixed in 4% PFA overnight, equilibrated in 30% sucrose overnight and embedded in OCT medium. Cryosections (18µm) were cut on a Reichert-Jung Frigocut 2800E and mounted on silane-coated slides. Before immunolabeling, cryosections were dried at room temperature, then incubated in cold methanol for 10 minutes, followed by 10 minutes incubation in 1%DMSO/1%TritonX-100/PBS. Primary and secondary antibodies, were applied using 1:200 and 1:1000 dilutions, respectively.

Additional feedings protocols

MIN and HFD were performed as described in Material and Methods. For 2xMIN feeding 0.75mg/larva Zebrafeed <100µm was fed twice per day. For 2xMIN+30mM Glucose 0.75mg/larva Zebrafeed <100µm was fed twice per day and 30mM glucose (Roth, 68887.1) was supplemented in the egg water.

2-NBDG uptake in zebrafish larvae

Pdx1 mutant larvae were HFD fed from 6 to 10 dpf. Larvae were treated with DMSO or 2µM Dorzagliatin from 6 to 10 dpf. From 9 to 10 dpf larvae were overnight incubated in 20µM 2-NBDG (MedChemExpress, HY-116215). 10 dpf larvae were anaesthesized with tricaine and embedded in 1.2% low melt agarose. The liver was imaged on a Zeiss Axio Observer.Z1 with a Yokogawa CSU-X1 spinning disk.

Supplemental Figures

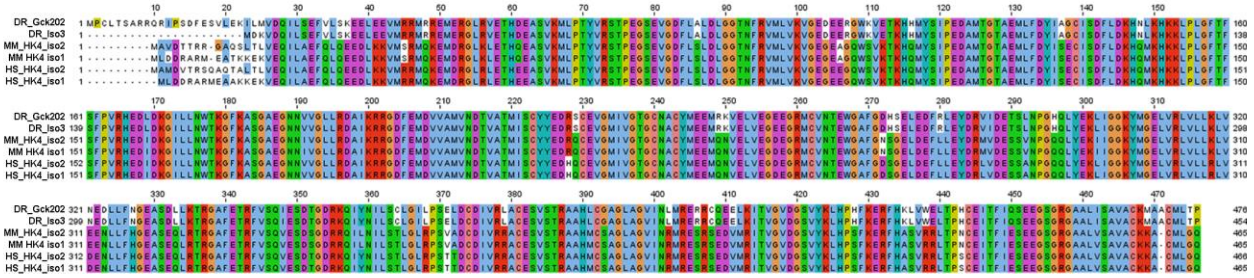

Supplemental Figure 1: Multiple sequence alignment of the main GCK isoforms. Amino acid alignment of the two main GCK isoforms in zebrafish (DR_Gck202 and DR_Iso3), mouse (MM_HK4_iso2 and MM_HK_iso1) and human (HS_HK4_iso2 and HS_HK4_iso1) showing high sequence similarity (more than 80%).

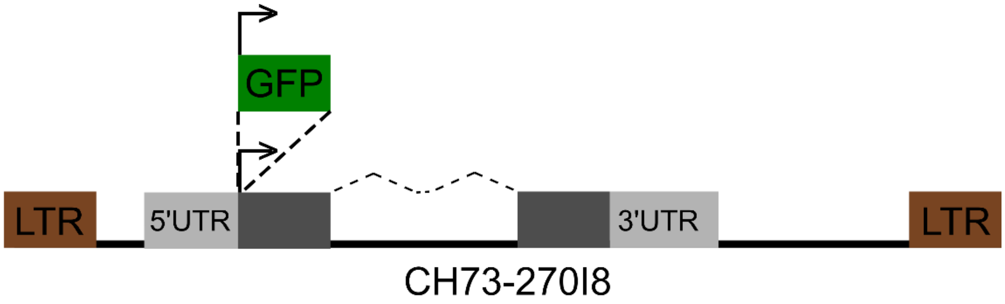

Supplemental Figure 2: Generation of the *Tg*$_{BAC}$*(gck:GFP)* reporter line. BAC CH73-270I8 was engineered to carry a GFP cassette downstream of the *gck201/202* ATG.

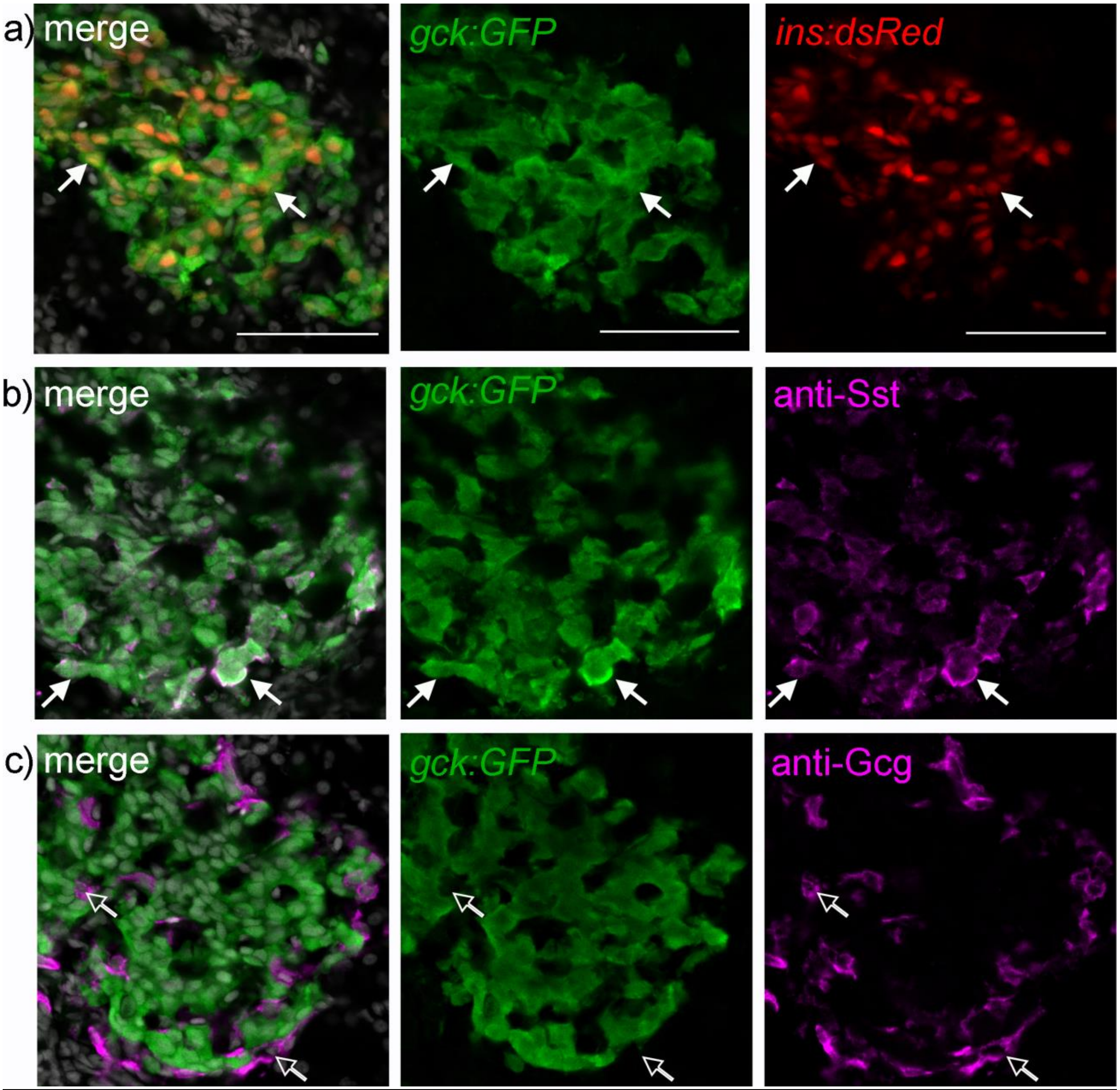

Supplemental Figure 3: *gck:GFP* recapitulates *gck* expression in adult islet cells. a) In adult fish all β-cells are co-labelled with dsRed and GFP (solid arrows), as observed in double transgenic *gck:GFP;ins:dsRed* animals. b) Immunohistochemistry revealed *gck:GFP* expression in all somatostatin labelled islet cells in adult fish. c) *gck:GFP* expression in glucagon expressing α-cells was rarely observed. Open arrows highlight hormone producing cells lacking *gck:GFP* staining. Scale bar: 100μm

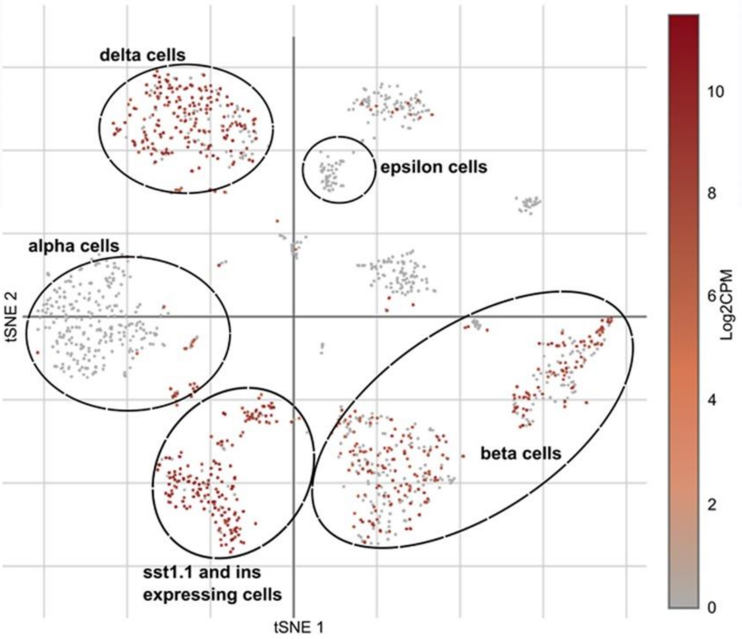

Supplemental Figure 4: tSNE plot visualizing *gck* expression in pancreatic islet cells (from ref 11) accessed via https://singlecell.broadinstitute.org/single_cell/study/SCP1549. Identity of hormone producing cells was annotated according to *glucagon*, *somatostatin*, *insulin* and *ghrelin* expression. Colors span a gradient from red (high expression) to grey (low expression).

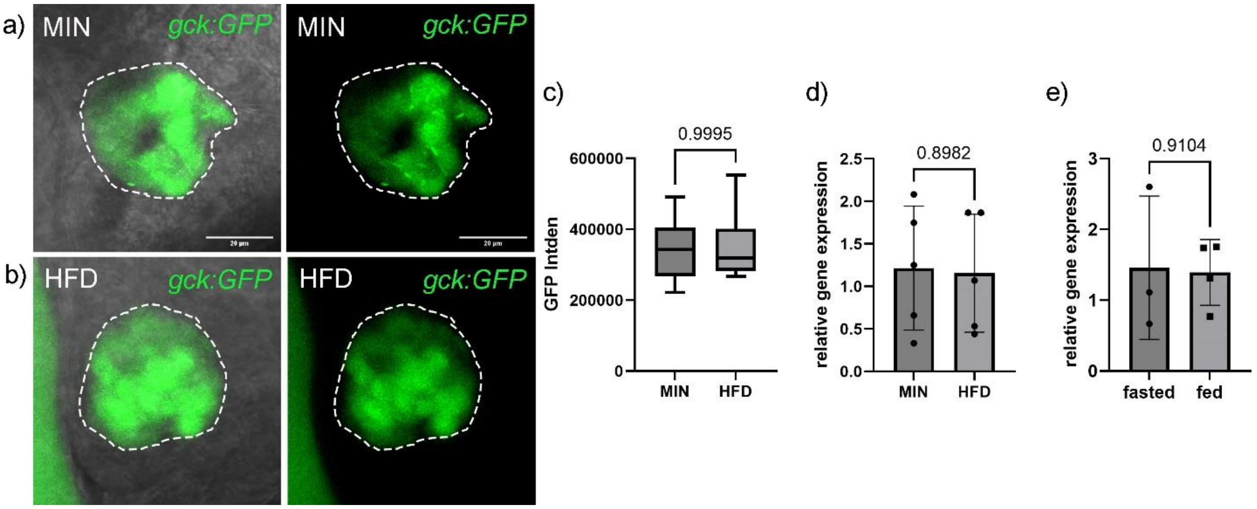

Supplemental Figure 5: Constitutive expression of *gck* in the pancreatic islets of zebrafish larvae and adults. *gck:GFP* expression in the endocrine islets (outlined) of larvae fed a MIN diet (a) or HFD (b) from 6 to 10 dpf shown as a maximun intensity projection. Scale bar: 20µm. c) Quantification of GFP signal in the endocrine islet of MIN and HFD fed larvae (N>9). Box plot with whiskers indicating Min to Max. Statistical significance assessed by t-test. d) *Gck* gene expression levels analyzed via RT-qPCR on isolated islets from larvae fed MIN and HFD from 6 to 10 dpf on larval islets (N=5). Scatter and bar plot shows mean with SD. Statistical significance assessed by t-test. e) *Gck* gene expression levels analyzed via RT-qPCR on dissected islets from fasted and fed adult fish (N>3). Scatter and bar plot shows mean with SD. Statistical significance assessed by t-test.

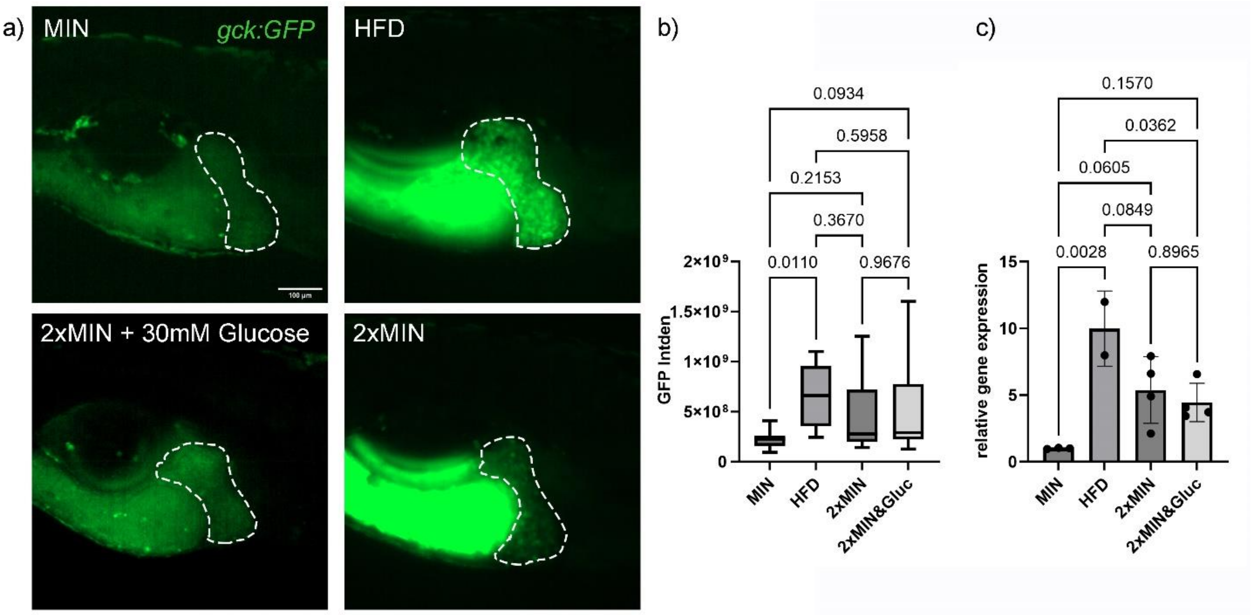

Supplemental Figure 6: Different feedings regimens affect *gck* expression. a) *Gck:GFP* expression in liver (outlined) of larvae fed from 6 to 10 dpf either one time MIN, HFD, two times MIN supplemented with 30mM Glucose or two times MIN per day. Scale bar: 100µm. b) Quantification of GFP signal in the liver of 10 dpf larvae fed different feeding regimens (N>12). Box plot with whiskers indicating Min to Max. Statistical significance assessed by One-way ANOVA. P-values of multiple comparisons are indicated. c) *Gck* gene expression levels analyzed via RT-qPCR in 10 dpf larvae fed different feeding regimens (N>2). Scatter and bar plot shows mean with SD. Statistical significance assessed by One-way ANOVA. P-values of multiple comparisons are indicated.

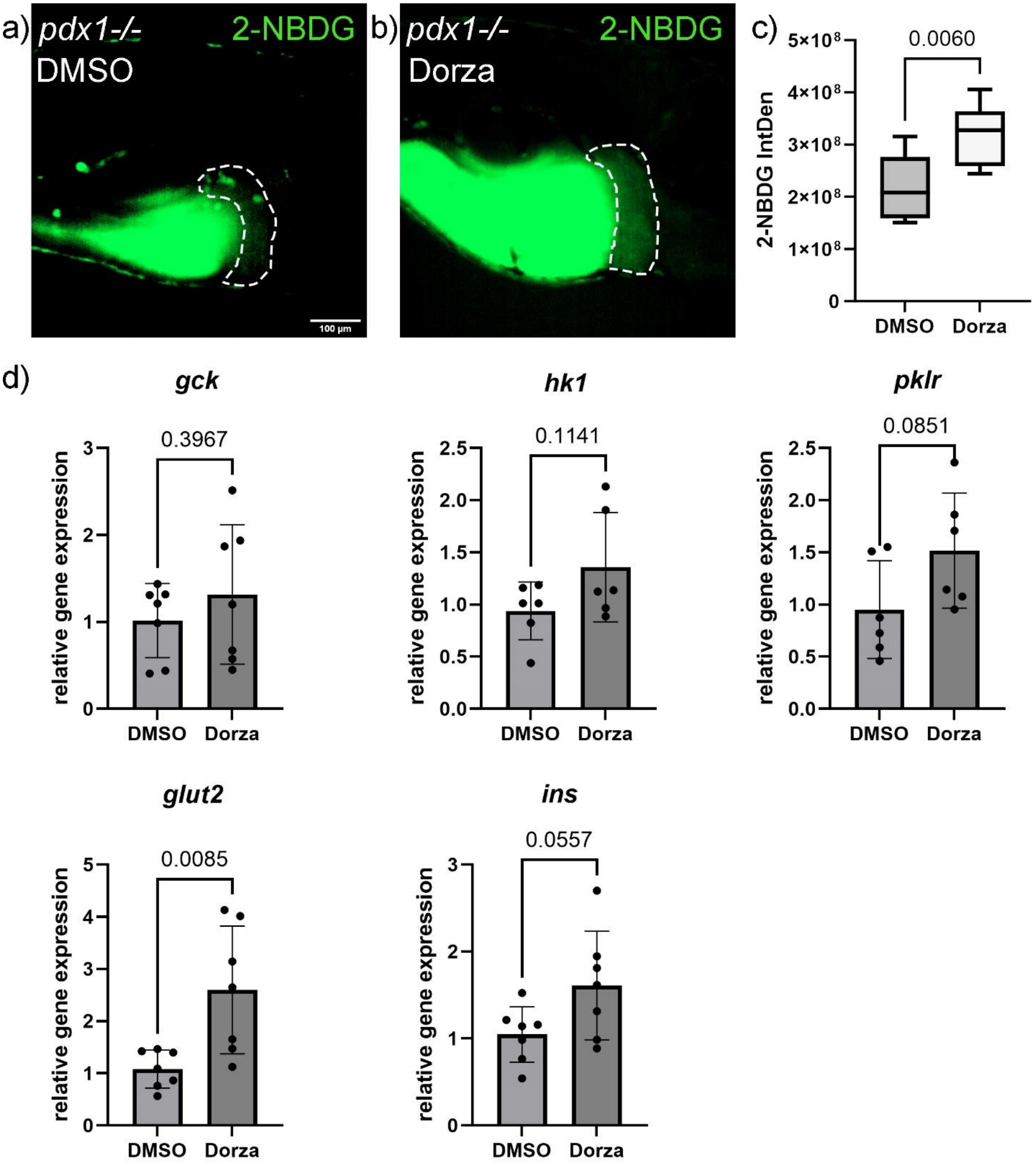

Supplemental Figure 7: Dorzagliatin affects glucose uptake and glucose metabolism in diabetic *pdx1* mutants. Uptake of the fluorescent glucose analogue 2-NBDG in the liver (outlined) of 10 dpf *pdx1* mutants fed HFD and treated with DMSO (a) or 2µM Dorzagliatin (b). Scale bar: 100µm. c) Quantification of fluorescent signal from 2-NBDG in the liver of DMSO and Dorzagliatin treated 10 dpf *pdx1* mutants (N>5). Box plot with whiskers indicating Min to Max. Statistical significance assessed by t-test. d) Relative expression levels of *gck*, *hk1*, *pklr*, *glut2* and *ins* assessed via RT-qPCR in 10 dpf *pdx1* mutant larvae, HFD fed, and either treated with DMSO or 2µM Dorzagliatin (N>6). Scatter and bar plot shows mean with SD. Statistical significance assessed by t-test.

References

1. Tarifeño-Saldivia, E. *et al.* Transcriptome analysis of pancreatic cells across distant species highlights novel important regulator genes. *BMC Biol.* **15**, 21 (2017).
2. Pozo-Morales, M. *et al.* In vivo imaging of calcium dynamics in zebrafish hepatocytes. *Hepatology* **77**, (2023).
3. Chen, S., Zhou, Y., Chen, Y. & Gu, J. fastp: an ultra-fast all-in-one FASTQ preprocessor. *Bioinformatics* **34**, i884–i890 (2018).
4. Lawson, N. D. *et al.* An improved zebrafish transcriptome annotation for sensitive and comprehensive detection of cell type-specific genes. *Elife* **9**, e55792 (2020).
5. Dobin, A. *et al.* STAR: ultrafast universal RNA-seq aligner. *Bioinformatics* **29**, 15–21 (2013).
6. Li, B. & Dewey, C. N. RSEM: accurate transcript quantification from RNA-Seq data with or without a reference genome. *BMC Bioinformatics* **12**, 323 (2011).
7. Li, H. *et al.* The Sequence Alignment/Map format and SAMtools. *Bioinformatics* **25**, 2078–2079 (2009).
8. Robinson, M. D., McCarthy, D. J. & Smyth, G. K. edgeR: a Bioconductor package for differential expression analysis of digital gene expression data. *Bioinformatics* **26**, 139–140 (2010).
9. Team, R. C. R: A Language and Environment for Statistical Computing. *R Found. Stat. Comput.* **Vienna**, **Au**, (2024).
10. Soneson, C., Love, M. I. & Robinson, M. D. Differential analyses for RNA-seq: transcript-level estimates improve gene-level inferences [version 2; peer review: 2 approved]. *F1000Research* **4**, (2016).
11. Singh, S. P. *et al.* A single-cell atlas of de novo β-cell regeneration reveals the contribution of hybrid β/δ-cells to diabetes recovery in zebrafish. *Development* **149**, dev199853 (2022).
