# Supplementary material for: Conserved glucokinase regulation in zebrafish confirms therapeutic utility for pharmacologic modulation in diabetes
Source: Commun Biol. 2024 Nov 23;7:1557. doi: 10.1038/s42003-024-07264-5 (PMC11585571; doi:10.1038/s42003-024-07264-5)
Supplement: Supplementary file 4 — Reporting summary [file 42003_2024_7264_MOESM4_ESM.pdf]

nature portfolio

Corresponding author(s): Nicole Schmitner
COMMSBIO-24-2250

Last updated by author(s): Nov 6, 2024

Reporting Summary

Nature Portfolio wishes to improve the reproducibility of the work that we publish. This form provides structure for consistency and transparency in reporting. For further information on Nature Portfolio policies, see our Editorial Policies and the Editorial Policy Checklist.

Statistics

For all statistical analyses, confirm that the following items are present in the figure legend, table legend, main text, or Methods section.

n/a	Confirmed	
☐	☒	The exact sample size (*n*) for each experimental group/condition, given as a discrete number and unit of measurement
☐	☒	A statement on whether measurements were taken from distinct samples or whether the same sample was measured repeatedly
☐	☒	The statistical test(s) used AND whether they are one- or two-sided<br>*Only common tests should be described solely by name; describe more complex techniques in the Methods section.*
☒	☐	A description of all covariates tested
☐	☒	A description of any assumptions or corrections, such as tests of normality and adjustment for multiple comparisons
☐	☒	A full description of the statistical parameters including central tendency (e.g. means) or other basic estimates (e.g. regression coefficient) AND variation (e.g. standard deviation) or associated estimates of uncertainty (e.g. confidence intervals)
☐	☒	For null hypothesis testing, the test statistic (e.g. *F*, *t*, *r*) with confidence intervals, effect sizes, degrees of freedom and *P* value noted<br>*Give P values as exact values whenever suitable.*
☒	☐	For Bayesian analysis, information on the choice of priors and Markov chain Monte Carlo settings
☒	☐	For hierarchical and complex designs, identification of the appropriate level for tests and full reporting of outcomes
☒	☐	Estimates of effect sizes (e.g. Cohen's *d*, Pearson's *r*), indicating how they were calculated

Our web collection on statistics for biologists contains articles on many of the points above.

Software and code

Policy information about availability of computer code

Data collection

Fluorescence images were acquired using Zen software. Image analysis was either performed with Imaris (Bitplane) or in ImageJ (as indicated in Material and Methods section).
RT-qPCR data was obtained using Bio-Rad CFX Manager.
Glucose measurements were obtained on a Victor® Plate Reader (PerkinElmer).
Glucokinase activity measurements were obtained on an EnSpire® Multimode Plate Reader (PerkinElmer).

Data analysis

- Quantifications:
Islet cell number quantification was performed with Imaris (Bitplane) using the Spot Detection function with a spot diameter of 4µm in 3D visualizations of confocal z-stacks spanning the region.
Quantification of fluorescence signal in islet and liver was quantified in ImageJ by making a Z projection using the 'Sum Slices' option.
- Glucokinase sequence analysis:
Gck annotations were compared in the integrated genome viewer (IGV.org).
Raw RNASeq reads from https://doi.org/10.1186/s12915-017-0362-x and 10.1002/hep.32663 were aligned against the zebrafish genome (GRCz11) using STAR.
Modified transcript annotations from v4.3.219 were used for gene and isoform level quantification using RSEM and summarized using tximport. Library size normalized counts were obtained using edgeR. Relative isoform usage for each sample was quantified directly via RSEM.
- Data and Statistics:
Data were processed using Excel2016 and Graphpad Prism10.2.0.
All data were analyzed and graphs were generated using Graphpad Prism10.2.0.

For manuscripts utilizing custom algorithms or software that are central to the research but not yet described in published literature, software must be made available to editors and reviewers. We strongly encourage code deposition in a community repository (e.g. GitHub). See the Nature Portfolio guidelines for submitting code & software for further information.

Data

Policy information about availability of data

All manuscripts must include a data availability statement. This statement should provide the following information, where applicable:

- Accession codes, unique identifiers, or web links for publicly available datasets
- A description of any restrictions on data availability
- For clinical datasets or third party data, please ensure that the statement adheres to our policy

All source data underlying the graphs presented in the Figures and Supplementary Figures are uploaded as Supplementary Data. All other data are available from the corresponding author upon reasonable request.

Human research participants

Policy information about studies involving human research participants and Sex and Gender in Research.

Reporting on sex and gender	N/A
Population characteristics	N/A
Recruitment	N/A
Ethics oversight	N/A

Note that full information on the approval of the study protocol must also be provided in the manuscript.

Field-specific reporting

Please select the one below that is the best fit for your research. If you are not sure, read the appropriate sections before making your selection.

☒ Life sciences ☐ Behavioural & social sciences ☐ Ecological, evolutionary & environmental sciences

For a reference copy of the document with all sections, see nature.com/documents/nr-reporting-summary-flat.pdf

Life sciences study design

All studies must disclose on these points even when the disclosure is negative.

Sample size	No statistical method was used to predetermine the sample size. Sample sizes were chosen based on accepted standards in the field.
Data exclusions	No data were excluded from analysis.
Replication	- For RNA extraction three to five larvae were pooled. At least three biological samples for each genotype and treatment were measured in two technical replicates.<br>- For glucose measurements three to five larvae were pooled for each sample. Four biological replicates for each genotype and treatment were measured in two technical replicates.<br>- For glucokinase activity measurements tissue extracts were prepared by homogenization of pooled larvae (4 to 11). At least three biological replicates for each genotype and treatment were measured in two technical replicates.<br>- For graphs analysing images, scatter dot plots were used for representation to confer sample size.
Randomization	Embryos were randomly collected from natural mating of zebrafish. To guarantee reproducibility and avoid bias, the larvae in an experiment came from pooled batches of eggs and experiments were repeated at least two times.<br>For experiments using mutants and feedings, the genotype was unknown prior to the experiments.<br>For experiments using mutants and drug treatments, the genotype was determined prior to the experiments.
Blinding	For feeding experiments using pdx1 mutants, wild-type and mutant animals were identified by genotyping after counting of endocrine cells and measurement of gck expression was performed. Image analysis and quantification were performed blindly.<br>For drug treatment experiments using pdx1 mutants, wild-type and mutant animals were identified by genotyping before experimental procedures to reduce the number of treated larvae. Experiments were not performed blindly. Image analysis and quantifications were performed blindly.

Reporting for specific materials, systems and methods

We require information from authors about some types of materials, experimental systems and methods used in many studies. Here, indicate whether each material, system or method listed is relevant to your study. If you are not sure if a list item applies to your research, read the appropriate section before selecting a response.

Materials & experimental systems

n/a	Involved in the study
☐	☒ Antibodies
☒	☐ Eukaryotic cell lines
☒	☐ Palaeontology and archaeology
☐	☒ Animals and other organisms
☒	☐ Clinical data
☒	☐ Dual use research of concern

Methods

n/a	Involved in the study
☒	☐ ChIP-seq
☒	☐ Flow cytometry
☒	☐ MRI-based neuroimaging

Antibodies

Antibodies used

Rabbit anti-somatostatin (Dako, A0566)
Mouse anti-glucagon (Sigma-Aldrich, G2654)
Chicken Anti-GFP antibody (Aves Labs, GFP-1010)
Goat anti-Chicken, Alexa Fluor Plus 488 (Invitrogen, A32931)
Goat anti-Mouse Alexa Fluor 546 (Invitrogen, A-11030)
Goat anti-Rabbit Alexa Fluor 568 (Molecular Probes, A-11011)

Validation

All antibodies used in this study were commercially available and have been used in previous studies.
Chicken Anti-GFP antibody (Aves Labs, GFP-1010) - Reference: https://doi.org/10.1038/s41467-024-47430-1
Rabbit anti-somatostatin (Dako, A0566) - https://zfin.org/ZDB-ATB-120207-3#summary
Mouse anti-glucagon (Sigma-Aldrich, G2654) - https://zfin.org/ZDB-ATB-081124-4

Animals and other research organisms

Policy information about studies involving animals; ARRIVE guidelines recommended for reporting animal research, and Sex and Gender in Research

Laboratory animals

Zebrafish were kept in a mixed Tuebingen and Mitfab692/b692/ednrb1b140/b140 background.

Wild animals

No wild animals were used in this study.

Reporting on sex

Sex was not considered in study design, as zebrafish sex cannot be determined at early developmental stages.

Field-collected samples

No field-collected samples were used in this study.

Ethics oversight

All procedures were approved by the Austrian Bundesministerium für Wissenschaft und Forschung (GZ BMWFW-66.008/0018-WF/V/3b/2017, GZ. 2020-0.282.289).

Note that full information on the approval of the study protocol must also be provided in the manuscript.
